# Total Arsenic and Arsenic Species Determination in Freshwater Fish by ICP-DRC-MS and HPLC/ICP-DRC-MS Techniques

**DOI:** 10.3390/molecules24030607

**Published:** 2019-02-09

**Authors:** Izabela Komorowicz, Adam Sajnóg, Danuta Barałkiewicz

**Affiliations:** Department of Trace Element Analysis by Spectroscopy Methods, Faculty of Chemistry, Adam Mickiewicz University in Poznań, 89b Umultowska Street, 61-614 Poznań, Poland; adam.sajnog@amu.edu.pl (A.S.); danutaba@amu.edu.pl (D.B.)

**Keywords:** arsenic, speciation, freshwater fish, ICP-DRC-MS, HPLC/ICP-DRC-MS

## Abstract

Analytical methods for the determination of total arsenic (TAs) and arsenic species (arsenite—As(III), arsenate—As(V), monomethylarsenic acid—MMA, dimethylarsenic acid—DMA and arsenobetaine—AsB) in freshwater fish samples were developed. Inductively coupled plasma mass spectrometry with dynamic reaction cell (ICP-DRC-MS) and high-performance liquid chromatography hyphenated to ICP-DRC-MS were used for TAs and arsenic species determination, respectively. The DRC with oxygen as a reaction gas was used. Sample preparation, digestion, and extraction were optimized. Microwave assisted digestion and extraction provided good recovery and extraction efficiency. Arsenic species were fully separated in 8 min using 10 mmol L^−1^ of ammonium dihydrogen phosphate and 10 mmol L^−1^ of ammonium nitrate. Overlapping of AsB and As(III) of arsenic species in the presence of a high concentration of AsB and trace amounts of As(III) were studied. Detailed validation of analytical procedures proved the reliability of analytical measurements. Both procedures were characterized by short-term and long-term precision: 2.2% (TAs) up to 4.2% (AsB), and 3.6% (TAs) up to 7.2% (DMA), respectively. Limits of detection (*L*_D_) were in the range from 0.056 µg L^−1^ for TAs to 0.15 µg L^−1^ for As(V). Obtained recoveries were in the range of 85%–116%. Developed methods were applied to freshwater fish samples analysis.

## 1. Introduction

Studies on arsenic speciation in freshwater fish are much less well known in comparison to arsenic speciation analysis in seawater samples, which is extensively researched and attracts greater attention [1,2]. In marine organisms, inorganic arsenic may be bioconverted to methylated species, such as monomethylarsenic acid (MMA) or arsenobetaine (AsB) [3]. AsB is the most often the end product of As metabolism, so it predominates. The biotransformation rate depends on intake of arsenic and transformation mechanisms in animals. Literature on freshwater fish generate opposite conclusions. Some papers report less concentration of total arsenic (TAs) in freshwater fish samples, while some report high levels of TAs and large proportions of inorganic arsenic [4,5]. Some authors assert that in freshwater fish samples, AsB is a major species, however, others suggest that freshwater fish may be a significant source of oxo-arseno-sugars or inorganic arsenic, but also arsenolipids, arsenocholine (AsC), 4-nitrophenylarsonic acid (NIT), 4-hydroxy-3-nitrobenzenearsonic acid (ROX), 4-aminobenzenearsonic acid (ASA), carbarsone (CA), p-hydroxyphenylarsonic acid (NAPP), trimethylarsine oxide (TMAO), or tetramethylarsonium (TMA) [5,6,7,8,9].

In order to distinguish differences in the toxicity, which demonstrate the investigated samples, it is necessary to perform speciation analysis, as determination of only TAs does not provide sufficient information about the toxicity of the sample. In the context of food analysis, determination of toxic arsenic species, mainly inorganic forms, such as arsenite (As(III)) and arsenate (As(V)), and organic methylacids, such as MMA and dimethylarsenic acid (DMA), is essential. It is also important to include AsB, a form that is frequently present in fish; however, AsB is considered a non-toxic organic arsenic compound which is rapidly excreted in unchanged form by humans [5]. On the other hand, the inorganic forms of arsenic, namely As(III) and As(V), are classified by the International Agency for Research on Cancer (IARC) as group 1 carcinogens with sufficient evidence of carcinogenicity in humans [10]. Chronic oral exposure to low levels of inorganic arsenic may cause dermal effects and peripheral neuropathy and also increased risk of skin cancer, lung, and bladder cancer. Oral exposure to MMA and DMA may result in gastrointestinal, kidney, and bladder damage [11]. The sources of arsenic in the natural waters are of anthropological origin, e.g., mining and smelting activity, herbicides and pesticides, waste incineration, and fuel combustion, or of natural origin, e.g., volcanic activity or leaching from rocks and minerals to groundwaters [5,12]. In view of the fact that freshwater fish are a highly popular food and part of the diet in some regions, due to their supplies of high value proteins and essential fatty acids, the consumption of arsenic contaminated fish may increase the exposure to its toxic species [12,13]. Arsenic is an element that is accumulated by organisms in a trophic chain to a considerable degree, from algae and plankton to the predatory fish, which are eventually consumed by people [14]. Therefore, arsenic speciation analysis in freshwater fish is an issue of great importance. However, in order to successfully assess the human health risk from organic and inorganic arsenic exposure, the appropriate standards and certified reference materials (CRM) with arsenic species must be developed, and complex data on arsenic speciation in food must be gathered, as well as further study on toxicity and metabolic processes and kinetics of arsenic species [14]. Furthermore, the development of new procedures for the hyphenated instrumental techniques and the recognition of the population health issues by legislators at national and international levels are crucial for proper evaluation of hazards concerning arsenic in food.

Speciation analysis of arsenic involves three steps: extraction, separation, and detection. A combination of analytical techniques must be used to obtain sufficient selectivity and sensitivity for speciation analysis. The concentrations and ratios of speciation forms in a sample should maintain unchanged during the preparation and extraction steps, so it is of critical importance to properly develop the analytical procedure. Various methods are applied to realize this purpose. Among them, the most often utilized hyphenated technique is high performance liquid chromatography inductively coupled plasma mass spectrometry (HPLC/ICP-MS) [12,15,16,17]; some authors have used other detection techniques, such as inductively coupled plasma optical emission spectrometry (ICP-OES), hydride generation atomic absorption spectrometry (HG-AAS), hydride generation atomic fluorescence spectrometry (HG-AFS), or electrothermal atomic absorption spectrometry (ET-AAS) [4,18,19]. In order to minimize spectral interferences hampering arsenic determination, the dynamic reaction cell (DRC) is often used in ICP-MS instruments [20,21]. The purpose of DRC is the elimination of polyatomic interferences forming in the plasma by introducing the reacting gas, usually ammonia, hydrogen, oxygen, or methane. Argon in plasma readily forms polyatomic ions, with the same mass to charge (*m*/*z*) as the analysed element, with matrix atoms in the sample. The reacting gas overcomes this by atom transfer or charge transfer reactions, thus breaking the polyatomic ion to atoms or ions with different *m*/*z*. Alternatively, the recommended approach to ^75^As quantification is the introduction of oxygen gas thus forming a new polyatomic ion ^91^AsO^+^, which is free from interfering species with *m/z* = 75 formed by chlorides and argon—^75^ArCl^+^. The most critical step in speciation analysis of solid samples is sample preparation. Many solvents and extraction methods for arsenic species from food samples have been tested. Methanol, water, a mixture of water/methanol in different ratios, and diluted acids are the most frequently used solvents. The most common extraction methods are mechanical stirring, Soxhlet extraction, ultrasound extraction, and microwave-assisted extraction [12,15,16,17,22].

The aim of this work was to establish methods for the determination of total arsenic and arsenic species in freshwater fish by ICP-DRC-MS and HPLC/ICP-DRC-MS techniques, respectively. Hence, the study was focused on digestion, extraction, separation, determination, and validation, which resulted in an accurate determination of TAs and arsenic species in freshwater fish samples. Special effort was taken to study the effects of overlapping of AsB and As(III). It is crucial to separate these two signals in the presence of a high concentration of AsB and trace amount of As(III). Procedures were applied to freshwater fish collected from the Wielkopolska and Lower Silesia provinces located in Poland.

## 2. Results and Discussion

### 2.1. Total Arsenic Determination

Arsenic was determined as an ^75^As^+^ ion and as ^91^AsO^+^, without and with use of DRC, respectively. Parameters responsible for the proper working of the DRC, such as, reaction gas flow rate, Rpq, and Rpa were optimized. Rpq and Rpa are the bandpass parameters, they set the appropriate RF and DC voltage of quadrupole rods, respectively. Reaction gas flow rate, Rpq, and Rpa were optimized in order to obtain the highest intensity for ^91^AsO^+^ in solution containing 10 µg L^−1^ of As and the lowest intensity in blank. Blank and standard solutions were prepared in three different ways: (a) with the use of distilled water and nitric acid, (b) with the use of fish matrix after digestion, (c) with the use of fish matrix after extraction. Reaction gas flow rate, Rpq, and Rpa were optimized thrice, as we had three pairs of blanks and standard solutions: (a), (b), and (c). The results did not differ significantly. The greatest difference in signal intensities between blank and standard solution was obtained at 0.55 mL min^−1^ gas flow rate, thus this flow rate was selected as optimal. Rpq and Rpa optimal values were 0.55 and 0, respectively.

Both ions were also monitored with and without ^74^Ge^+^ and ^103^Rh^+^ internal standards in order to check if non-spectral interferences appear. The main purpose of this experiment was to choose the best mode for TAs determination. It was checked by analysis of certified reference materials (CRMs): Tuna Fish Tissue BCR-627; Fish Muscle ERM-BB422; Herring Tissue MODAS-3, and Cod Tissue MODAS-5, previously digested according to the procedure described in Materials and Methods. The results obtained for digested samples for Tuna Fish Tissue BCR-627 and Fish Muscle ERM-BB422 are presented in Table 1 They indicate that the best mode for TAs determination is to monitor ^91^AsO^+^ ion with the use of DRC without an internal standard.

### 2.2. Extraction of Arsenic Species from Freshwater Fish Samples

Extraction is a crucial step in speciation analysis of solid samples. It is necessary to achieve good extraction efficiency as we deal with trace analysis and maintain species equilibrium at the same time. Application of the least aggressive reagents and extraction conditions may prevent species interconversion during extraction.

**Figure 1:**
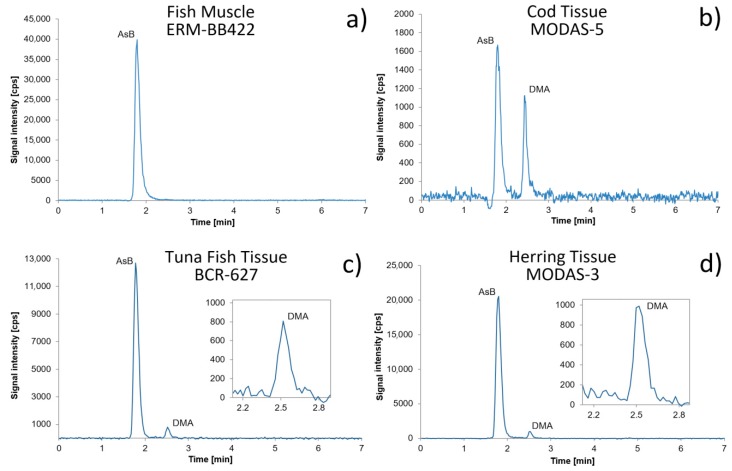
Chromatograms of (**a**) Fish Muscle ERM-BB422, (**b**) Cod Tissue MODAS-5, (**c**) Tuna Fish Tissue BCR 627, and (**d**) Herring Tissue MODAS-3.

Samples were submitted to ultrasonic and microwave assisted extraction (ME). Ultrasonic bath (UB) was significantly less effective than the ultrasonic probe (UP) and ME methods. Extraction efficiencies were evaluated by the analysis of the above-mentioned CRMs. A mass balance of As was calculated by comparing the sum of the concentration of extracted species with the TA concentration determined in the extracts. Obtained extraction efficiencies were about 20% worse than these obtained using ME. Application of UP enabled extraction efficiencies to be achieved, which were about 10% worse than those obtained with ME, which is in agreement with results obtained by other authors [17]. With regard to this, our further efforts were focused on ME. The results obtained for microwave extracted samples of CRMs confirmed that the best mode for arsenic analysis is to monitor the ^91^AsO^+^ ion with use of DRC without an internal standard (Table 2). Therefore, in subsequent studies arsenic was determined as ^91^AsO^+^ with DRC. Significantly better recoveries, ranging from 92% to 99% for all used CRMs, were obtained when the samples were extracted with water in comparison to water-methanol solution 1:1. These results are in good agreement with those published previously, where using pure water as extracting agent provided the best extraction efficiency in comparison to water methanol solutions [16,23,24]. The results obtained with water extraction and extraction with water-methanol solution 1:1 are presented in Table 1b. Extraction efficiencies obtained for water-methanol solution 1:2 and water-methanol solution 1:3 were about 20%–30% worse than those obtained for water extracted samples; they decreased with an increase of concentration of methanol in the extracting solution, thus they are not presented. In further studies, samples were extracted solely with water. Chromatograms of all CRMs after water extraction are shown in Figure 1. There are reports in the literature that extraction efficiency of As species are much less than 100%, often in the range of 40%–60% when anion-exchange column was used [5,25,26], or 16%–42% for reversed-phase columns [8]. This was occurring more readily when inorganic As species were present in higher concentrations in an analyzed sample or when unknown As species were also indicated in the chromatograms.

### 2.3. Chromatographic Separation of Arsenic Species

Arsenic species separation was conducted using anion-exchange chromatography. We used our previously developed procedure dedicated to arsenic speciation analysis in water samples [27]. pH value was slightly modified, the optimal value was set at 9.1. Using these chromatographic conditions, the retention times for all species were in the range from 1.8 min to 6.9 min. The order of elution was AsB, As(III), DMA, MMA, and As(V). Chromatographic separation expressed by resolution was calculated with use of retention times and base signal widths (calculated as a time) for analyte signals. Resolution values of the analytical column, calculated for neighbouring signals of AsB-As(III), As(III)-DMA, DMA-MMA, and MMA-As(V), were 1.3, 1.3, 7.3, and 5.8, respectively.

Additionally, we applied other separation conditions to obtain better resolution of AsB and As(III), which involved the application of 20 mmol L^−1^ of ammonium carbonate at pH 8.6. Complete separation of these two species was obtained within 4 min. The obtained AsB-As(III) resolution was equal to 1.5.

### 2.4. Figures of Merit

#### 2.4.1. Total Arsenic

The calibration curve for TAs was constructed in the concentration range of 0.2 µg L^−1^ to 50 µg L^−1^. Three equidistant calibration standards for each calibration point were made and the average value for each standard was taken in order to construct the calibration curve. The limit of detection (*L*_D_) was determined by analysis of 10 independent samples of the same freshwater fish sample after digestion (according to the digestion programme described in chapter 3 Materials and Methods) and twice diluted (to obtain freshwater fish matrix, without detectable amounts of analyte) with addition of TAs in the concentration of 0.5 µg L^−1^. The *L*_D_ value was calculated according to the equation:

*L*_D_ = 3*sd*, (1)

where *sd* denotes the standard deviation of the measurements. Limit of quantification (*L*_Q_) was calculated according to the equation:

*L*_Q_ = 3*L*_D_, (2)

Repeatability was calculated on the basis of the analysis of three individually prepared CRM Tuna Fish Tissue BCR-627 samples (after digestion) and one freshwater fish sample (after digestion). Each sample was analyzed 3 times, therefore repeatability calculation was based on 12 digests. Short-term repeatability was calculated within a day; however, long-term repeatability was calculated for three weeks, repeating measurements every week. Obtained short-term and long-term precision values were equal to 2.2% and 3.6%, respectively. To evaluate the trueness of the method, the following CRMs were analysed: Tuna Fish Tissue BCR-627; Fish Muscle ERM-BB422; Herring Tissue MODAS-3; and Cod Tissue MODAS-5. The results of TAs obtained from the analysis of digested CRMs were compared to its certified values, in particular CRM and the Student’s *t*-test was used to verify any significant differences between these values. The Student’s *t*-test at a 95% confidence level showed good agreement of the results. Detailed characteristics of the developed analytical procedure are presented in Table 3.

#### 2.4.2. As Species

**Figure 2:**
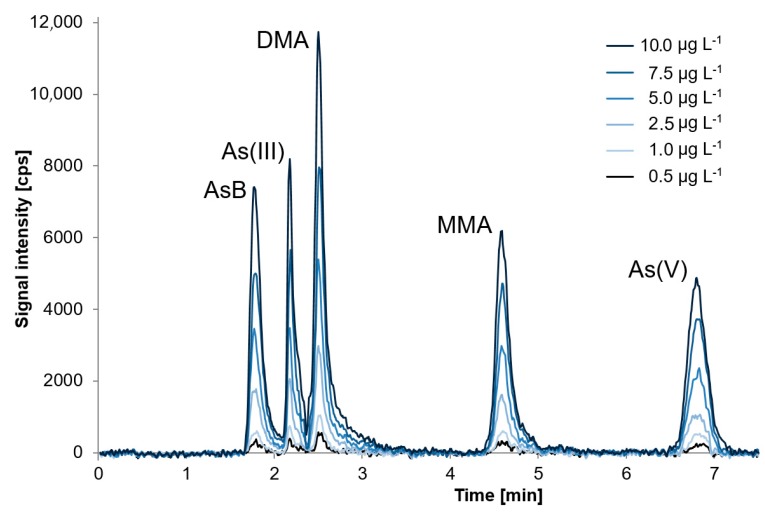
Chromatographic separation obtained for analytes in standards (taken to construct calibration curves) in the range of: 0.5 µg L^−1^ to 10.0 µg L^−1^ obtained using a mixture of 10 mmol L^−1^ of ammonium phosphate and 10 mmol L^−1^ of ammonium nitrate as mobile phase.

Calibration curves for AsB, As(III), As(V), DMA, and MMA were constructed in the concentration range of 0.5 µg L^−1^ to 10 µg L^−1^. Chromatographic separation obtained for analytes in standards in mentioned concentration ranges is presented in the Figure 2. Three independent solutions of calibration standards were measured for each calibration point, the average value for each standard was taken in order to construct the calibration curves. Snedecor’s *F*-test confirmed that the concentration ranges of the calibration curves were chosen correctly as variances of the lowest and the highest standard concentration of AsB, As(III), DMA, MMA, and As(V) are homoscedastic. In order to determine *L*_D_, 10 independent samples of the same freshwater fish sample after extraction, twice diluted and with addition of all analytes in the concentration of 1 µg L^−1^, were prepared and analysed using the developed analytical procedure. *L*_D_ and *L*_Q_ values were calculated as described above for TAs. For calculation of repeatability, 12 independent determinations of AsB, As(III), DMA, MMA, and As(V) were conducted using two individually prepared Tuna Fish Tissue BCR-627 and two individually prepared freshwater fish samples (*n* = 3 for each sample), both spiked with mixed standard solution, containing all investigated species in the concentration of 1 µg L^−1^ and 5 µg L^−1^ and then extracted in conditions presented in chapter 2.2. Short-term and long-term repeatability were calculated in the same way as for TAs. Short-term repeatability was in the range of 3.3% to 4.2% and long-term repeatability was in the range of 4.2% to 7.2%. Due to the unavailability of certified reference materials with certified values for all investigated species, trueness was mainly verified using the standard addition method. However, in the case of AsB and DMA, trueness was verified directly by the analysis of: Fish Muscle ERM-BB422; Herring Tissue MODAS-3, Cod Tissue MODAS-5, and Tuna Fish Tissue BCR-627 after extraction. The same samples as for repeatability calculation, Tuna Fish Tissue BCR-627, and a freshwater fish sample after extraction, were spiked with mixed standard solution, as described above for repeatability calculation. On that basis, trueness for other species was checked. Samples without spikes were analyzed without dilution to determine DMA which occurred at trace level, and after dilution to determine AsB. Values for recoveries calculated after direct analysis were in the range of 89% to 103% (details in Table 4), and those obtained using the standard addition method were in the range of 85% to 116%. Detailed characteristics of the developed analytical procedure are presented in Table 4 and Table 5. Figure 1 presents chromatograms obtained for all CRMs.

### 2.5. Study on the Effect of Overlapping AsB and As(III) Analytical Signals

**Figure 3:**
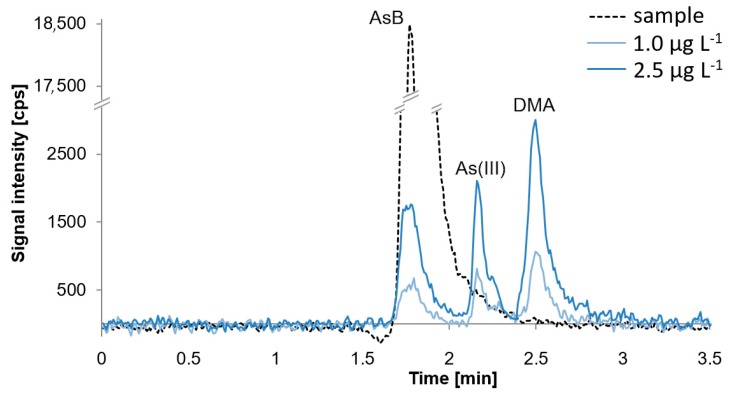
Chromatogram presenting selected fish sample with the concentration of AsB equal to 4.5 µg g^−1^ (35 µg L^−1^) and standards with the concentration of 1.0 µg L^−1^ and 2.5 µg L^−1^ obtained using a mixture of 10 mmol L^−1^ of ammonium phosphate and 10 mmol L^−1^ of ammonium nitrate as mobile phase.

Ion chromatography is an attractive technique for elemental speciation analysis, as it may separate charged species. All species of interest, with the exception of AsB, have appropriate dissociation constants to be separated as anions in alkaline solution using ion chromatography [28]. AsB in alkaline conditions exists as a zwitterion, hence it is eluted first. Using the described separation conditions signals from AsB, As(III) and DMA elute at the beginning of the analysis. They are separated with good resolution, although pH value must be rigorously kept constant during experiments, as any slight change may result in co-elution of AsB and As(III) or As(III) and DMA. We confirmed, by the calibration, that AsB, As(III), and DMA may be determined in the range of 0.5 µg L^−1^ to 10 µg L^−1^. Quantification would not be a problem when species occur in samples at the same level. However, with regard to fish samples which may in predominance contain AsB, it is difficult to separate AsB and As(III) if the concentration of AsB is high and the concentration of As(III) is at trace level. As was mentioned in the Introduction, this is especially problematic in freshwater fish samples, in contrast to marine fish. Freshwater fish also contain toxic arsenic species like As(III) and As(V), sometimes at trace levels but in some cases also as predominant species when they come from areas contaminated with arsenic. In the first case, a high signal of AsB overlaps the As(III) signal; it may overlap the As(III) signal entirely when As(III) occurs below 1 µg L^−1^ and the signal of AsB is appropriately high. Figure 3 presents a chromatogram of Tuna Fish Tissue BCR-627 and standard solutions in the concentration of 1.0 µg L^−1^ and 2.5 µg L^−1^.

Overlapping the signal of As(III) by AsB is demonstrated; the concentration of AsB is about 35 µg L^−1^ (4.5 µg g^−1^). In these conditions, it would be possible to determine As(III) from a concentration of 2 µg L^−1^.

**Figure 4:**
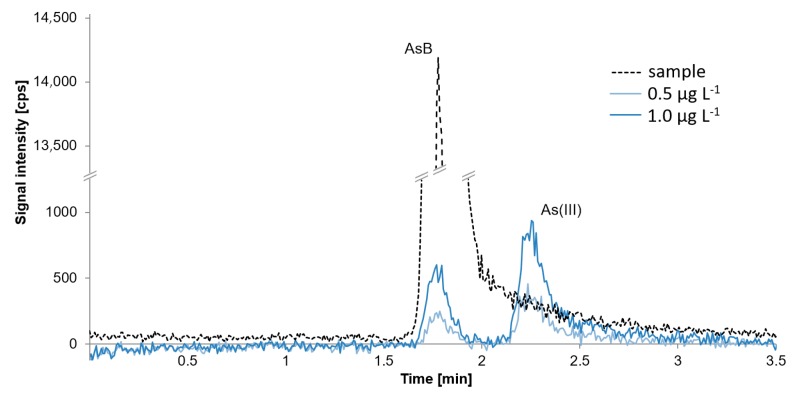
Chromatogram presenting selected fish sample with the concentration of AsB equal to 4.5 µg g^−1^ and standards with the concentration of 0.5 µg L^−1^ and 1 µg L^−1^ obtained using 20 mmol L^−1^ of ammonium carbonate as mobile phase.

In order to obtain better separation between AsB and As(III) signals, we applied a different procedure with ammonium carbonate as the component of the mobile phase. Figure 4 presents a chromatogram of a freshwater fish sample containing only AsB confronted with chromatograms of standards in the concentration of 0.5 µg L^−1^ and 1 µg L^−1^. It may be observed that quantification of As(III) is impossible if it occurs in a concentration ≤0.5 µg L^−1^; the signal corresponding to 0.5 µg L^−1^ concentration is entirely covered by the AsB signal. Direct quantification using this procedure may be done when the concentration of As(III) is above 1 µg L^−1^.

**Figure 5:**
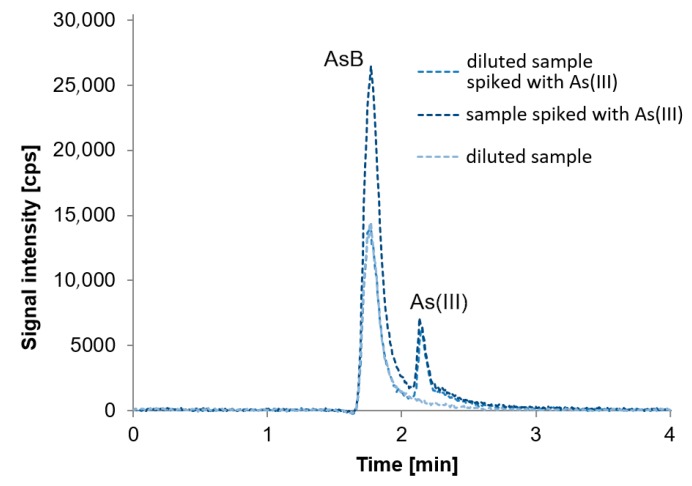
Chromatogram of freshwater fish sample: Undiluted spiked with As(III) with the concentration of 5 µg L^−1^, twice diluted spiked with As(III) with the concentration of 5 µg L^−1^, and twice diluted not spiked.

With regard to the above, in order to quantify the As(III) present at concentration below 1 µg L^−1^, the standard addition method is essential. The chromatogram of a freshwater fish sample (undiluted and twice diluted) spiked with As(III) at a concentration of 5 µg L^−1^ and a twice diluted sample not spiked is shown in Figure 5. Co-elution of AsB and As(III) is visible but the separation of the signal and their quantification is possible for the diluted sample as well as for the undiluted one.

### 2.6. Determination of Arsenic Species in CRM and Freshwater Fish Samples

Arsenic speciation analysis in CRM samples revealed, according to the chromatograms presented in Figure 1, the presence of AsB as a predominant species in Tuna Fish Tissue BCR-627, Herring Tissue MODAS-3, and Cod Tissue MODAS-5. The presence of DMA was confirmed. Only in Fish Muscle ERM-BB422 did arsenic occur just in the form of arsenobetaine. As(III), MMA, and As(V) were not observed in any CRM. CRM samples were analyzed directly in order to determine DMA concentration and after dilution in order to determine AsB concentration. The results for As species determination in different CRM are shown in Table 3.

**Figure 6:**
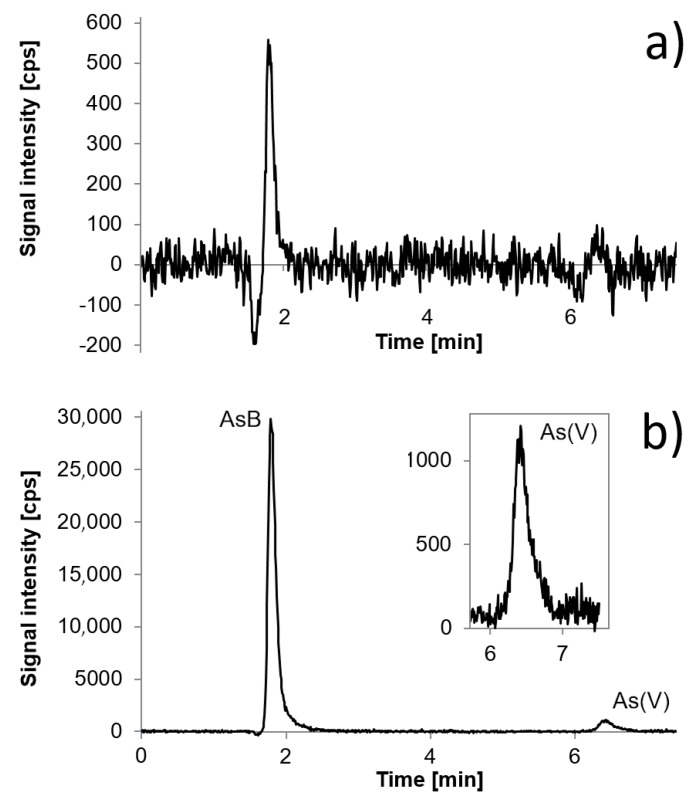
Chromatograms of exemplary freshwater fish samples: (**a**) from Wielkopolska province; (**b**) from Lower Silesia province.

Freshwater fish samples were collected from Lower Silesia and Wielkopolska provinces. Lower Silesia is a region in Poland which is specific with regard to arsenic presence, as the local geochemical structure includes deposits of arsenic [29]. According to literature reports, freshwater fish collected from areas rich in arsenic were able to bioaccumulate arsenic, not only as non-toxic AsB but also toxic As(III) or As(V). For this reason, the freshwater fish were collected from Lower Silesia. The concentrations of TA in the stream that feeds the water reservoirs in Lower Silesia where the fish were collected are reaching up to 3778 µg L^−1^, mainly in the form of As(V) [29]. In other river from this region, the concertation of TA reached 11 µg L^−1^, also mostly as As(V) species. The fish samples collected from Wielkopolska may be treated as control samples. The waters in Wielkopolska contained arsenic at much lower concentrations, from 0.10 to 1.8 µg L^−1^. All samples were digested according to the program described in Materials and Methods and analyzed using the ICP-DRC-MS technique. Samples from Lower Silesia contained up to 80 times more TAs than samples from Wielkopolska. The predominant species in all freshwater fish samples was AsB. In the samples from Wielkopolska, in which the presence of TAs was stated, HPLC/ICP-DRC-MS analysis confirmed that all arsenic was in the form of AsB at a very low concentration. The vast majority of samples from Lower Silesia also contained AsB. A trace amount of As(V) was stated; this form comprised from 0.72% to 3.34% of TAs. Results indicate that occurrence of concrete arsenic species do not depend on fish species but rather on the area they live in, as similar results were obtained for freshwater fish samples collected in Wielkopolska province and for freshwater fish samples collected in Lower Silesia province. Differences in concentration of particular species in fish collected from the same region of course exist, however age and weight of fish are also factors which have an influence on it. Extraction efficiency and column recovery were acceptable. The concentration of TAs and arsenic species in all the analyzed freshwater fish samples are shown in Table 5. Chromatograms of exemplary freshwater fish samples are presented in Figure 6.

Shah et al. ascertained the presence of As(III) and As(V) in edible fish species collected from an arsenic contaminated lake in Pakistan in the concentration range from 1.19 µg g^−1^ to 2.05 µg g^−1^ and from 0.17 µg g^−1^ to 0.46 µg g^−1^, respectively [4]. Also, Devesa et al. stated that AsB was not a predominant species in freshwater crustaceans obtained from areas affected by toxic spill from the mines, but inorganic arsenic (from 0.34 µg g^−1^ to 5.4 µg g^−1^) predominated. The authors found few arsenosugars and unknown arsenic species [30]. On the other hand, AsB was the main As species found in bivalve mollusks from Brazil in the paper where authors evaluated microwave and ultrasound radiation, combined with different extraction conditions. The best extraction efficiency for TA analysis was achieved by using pure water as an extracting solution, similar to the current study [17]. Ciardullo et al. determined 17 As species in 4 species of freshwater fish from river Tiber, using both anion-exchange and cation-exchange chromatography [26]. The most abundant As species was AsB (58%–96% of all As species in methanol/water extract) with traces of DMA, TMAO, and arseno-sugars, but also the unknown As species. Also, AsB was a predominant species in most fish samples collected from Xiang River in China, with a mean concentration of TA in fish muscle equal to 0.75 µg g^−1^ [12]. Authors noticed that the percentage of AsB (AsB%) in fish muscle was not relevant to TA concentration, while the percentage of inorganic As decreased with TA concentration in a hyperbolic pattern. This phenomena was explained by the restricted assimilation and accumulation of toxic inorganic As with increasing TA concentration in fish. This could also explain such high concentrations of AsB and very low concentrations of other As species in the current study. AsB was also the predominant As form in freshwater fish collected form Hayakawa River in Japan using reversed-phase column in the study described by Miyashita et al. [8]. Besides AsB, also other species were determined, e.g., DMA and oxo-arseno-sugars, however, their concentrations and ratios were species dependent. Interestingly, authors reported a large percentage of non-extractable As species, contributing more than 50% of the TAs concentrations, which suggests that the biochemistry of As in freshwater fish is more complex than in marine organisms. The concentration of TA in fish muscle of over 20 fish species form As contaminated ponds in Thailand was correlated with the As concentration in water [5]. The main As species found in fish muscles therein were DMA, AsB, and TMAO, however, it was dependent on the species of fish under evaluation. The study concludes that higher As concentrations in water results in roughly proportional elevation of As concentration in muscles of fish living in those waters. A similar tendency was found in the current study with regard to the As concentration in reservoirs form Wielkopolska and Lower Silesia reported previously [29]. Juncos et al. provided the conclusion that fish from lakes that are closer to the Puyehue-Cordón Caulle volcanic complex contain higher concentrations of As, mostly in the form of AsB and DMA, with no inorganic As species. However, the highest concentration of TA in muscles was 1.16 µg g^−1^ and significantly higher levels of As were determined in liver, kidney, and gills (up to 4.31 µg g^−1^) where the most metabolic reactions occur. [25]. The contradictory results for AsB determination in freshwater fish muscle and other organs were described by Yang et al., where no AsB was detected and the main species were DMA and MMA with small amounts of As(III), with highest concentrations found in the liver [31].

The results of some of the above described research give rise to a health concerns for people whose diet is based on freshwater fish, since many species of fish contained significant amounts of inorganic As, including the most toxic As(III) [5,12]. It was reported that the liver and stomach of fish (tilapia) play the most important role in absorption and accumulation of As, in contrast to the edible fish muscle with the bioconcentration factors for liver and stomach in the range 1.0–3.1 and for muscle in the range 0.2–0.3, which is an important and positive conclusion form the point of view of the general population’s health [32]. It was also found that concentration of As species in farmed fish in Hungary was roughly proportional to the content of As in fishmeal. However, the predominant species was AsB (5.0 µg g^−1^) and other forms were at trace levels, and therefore do not pose a toxicological concern [9].

## 3. Materials and Methods

### 3.1. Instrumentation

For TAs, all measurements were carried out using an Elan DRC II ICP-MS (PerkinElmerSCIEX, Vaughan, Ontario, Canada), equipped with a cyclonic spray chamber, a concentration glass nebulizer, and a quartz torch with a quartz injector. DRC, with oxygen as a reaction gas, was employed to remove spectral interferences. For arsenic speciation, HPLC/ICP-DRC-MS was used. The HPLC system consisted of a pump, an autosampler, and a column oven (PerkinElmer Series 200 or 225, PerkinElmerSCIEX, Vaughan, Ontario, Canada). The autosampler was equipped with a Peltier Cooling Tray used for maintaining samples at temperature of 4 °C, before and during analysis. Arsenic species were separated using the anion-exchange column PRP-X100 (4.6 mm × 150 mm) in PEEK material purchased from the Hamilton Company (Bonaduz, Switzerland).

The instrumentation was also equipped with an automatic switching valve (Rheodyne, Rohnet Park, CA, USA) that allowed operation between the HPLC and the ICP-DRC-MS sample introduction system. Data was collected using Chromera software (version 2.1.0.1631, PerkinElmerSCIEX, Vaughan, Ontario, Canada). The HPLC and ICP-MS operating conditions are presented in Table 6.

A microwave system (Ethos One, Milestone Srl, Sorisole, Italy) was used for the digestion of fish muscles and their extraction. An ultrasonic processor with a 3 mm titanium probe and an ultrasonic bath, both purchased from Bandelin (Berlin, Germany), were used for sample sonication. An electronic pH meter calibrated with three buffer solutions (pH 4.01, 6.87 and 9.18) was used for setting the pH of all mobile phases (WTW, Weilheim, Germany).

### 3.2. Reagents and Solutions

#### 3.2.1. Arsenic Working Solutions

Stock solution of As(III) was prepared from (1002 ± 5) mg L^−1^ As solution consisting of arsenic trioxide in a mixture of 0.3% sodium hydroxide, 0.5% (*v*/*v*) hydrochloric acid, and 0.06% sodium bicarbonate (Inorganic ventures, Christiansburg, Virginia). As(V) stock solution was prepared from (994 ± 6) mg L^−1^ As solution, which contained arsenic(V) oxide hydrate in water (Inorganic ventures, Christiansburg, Virginia, USA). DMA (a purity of 99%), MMA prepared from monosodium acid methane arsonate sesquihydrate (a purity of 98.5%), and AsB (a purity of 95%) were purchased from Sigma-Aldrich (Steinheim, Germany). Individual working solutions of As used for optimization or calibration purposes were prepared daily and stored at 4 °C in plastic vessels (flasks made of polymethylpentene, vials made of polypropylene) in the dark.

#### 3.2.2. Mobile Phase

Ammonium phosphate dibasic (a purity of 99.9999%), ammonium nitrate (a purity of 99.999%), and ammonium carbonate (puriss. p.a.), all purchased from Sigma-Aldrich (Steinheim, Germany), were used as mobile phase components. Methanol HPLC Gradient Grade with a purity of 99.8% used in the course of the optimization was purchased from J. T. Baker (Philipsburg, New Jersey, USA). Sodium hydroxide pellets (used as a 30% solution), Suprapur ammonia solution of 25% (*v*/*v*), and Suprapur nitric acid of 65% (*v*/*v*) used for pH adjustment were purchased from Merck (Darmstadt, Germany). The mobile phase was always filtered through a membrane filter with a pore size of 0.2 µm. Buffer solutions with pH equal to 4.01, 6.87, and 9.18 used for pH meter calibration were purchased from Schott Instruments (Mainz, Germany). Eluents were stored at 4 °C in plastic bottles.

#### 3.2.3. Certified Reference Materials

Tuna Fish Tissue BCR-627 and Fish Muscle ERM-BB422 (*Pollachius virens*, Saithe) purchased from the Joint Research Centre, Institute for Reference Materials and Measurements (Geel, Belgium), Herring Tissue MODAS-3 and Cod Tissue MODAS-5 purchased from the Institute of Nuclear Chemistry and Technology (Warsaw, Poland) were used for the optimization of digestion and extraction procedure and for quality control purposes.

#### 3.2.4. Other

Concentrated nitric acid (65% HNO_3_ Suprapur, Merck, Germany) and hydrogen peroxide (30% H_2_O_2_, Sigma-Aldrich, USA) were used for digestion purposes. Argon with a purity of 99.999% was used as a nebulizer, auxiliary, and plasma gas (Linde Gas, Kraków, Poland). High purity oxygen (Linde Gas, Kraków, Poland) was used as the DRC gas. Germanium and rhodium solutions at a concentration level of 10 µg L^−1^ were prepared from individual 1000 mg L^−1^ Ge and Rh stock solutions in 2% HNO_3_ (Merck, Darmstadt, Germany). Smart Tune Solution—ELAN DRC/PLUS/II was used to check the daily performance of ICP-DRC-MS. Distilled, deionized water (Direct-Q 3UV Water Purification System, Merck, Germany) was used throughout the experiments.

### 3.3. Samples and Sample Preparation

Freshwater fish samples of edible species, namely carp (*Cyprinus carpio)*, crucian carp (*Carassius carassius)*, silver bream (*Blicca bjoerkna)*, bream (*Abramis brama)*, trout (*Oncorhynchus mykiss*), and sturgeon (*Acipenser*), were collected from Wielkopolska and Lower Silesia provinces. Directly after collection, the fish were transported to the laboratory and were either frozen or underwent the procedure described below. Only edible parts of the fish samples were utilized for the analysis. Small pieces of fish muscles were cut from the fish body using a knife with a ceramic blade, washed with deionized water, and dried at 60 °C in a dryer with forced air circulation (SLN 32 STD, Pol-Eko, Wodzisław Śląski, Poland) until at a constant weight. The samples were then ground in a vibrating ball mill (Mini-Mill Pulverisette 23, FRITSCH, Idar-Oberstein, Germany) for 8 min.

#### 3.3.1. Digestion

The mass of 0.2–0.3 g of homogenized fish samples was added to digestion vessels with 2 mL of concentrated nitric acid and 0.5 mL of hydrogen peroxide. Prepared samples were placed into a microwave oven. The microwave digestion program was as follows: (1) ramp time 15 min, power 1500 W, temperature 180 °C; (2) hold time 20 min, power 1500 W, temperature 180 °C. After cooling down to room temperature, samples were quantitatively transferred to volumetric flasks and diluted with deionized water. Procedural blanks (one for each digestion run) were prepared in an acid matrix exactly the same as used for sample preparation. Digestion procedure was optimized using certified reference materials: Tuna Fish Tissue BCR-627; Fish Muscle ERM-BB422; Herring Tissue MODAS-3; and Cod Tissue MODAS-5. To avoid possible spectral interferences accompanying arsenic ICP-MS measurements, a dynamic reaction cell with oxygen as a reaction gas was used. The samples were analyzed in standard mode (without internal standard) and with ^74^Ge^+^ and ^103^Rh^+^ used as internal standards in order to eliminate possible non-spectral interferences. Arsenic was determined as an ^75^As^+^ ion and as ^91^AsO^+^. Procedural blanks and samples were prepared and analyzed in triplicate.

#### 3.3.2. Extraction

The following approaches: UB, UP, and ME, for sample extraction were used. A quantity of 0.2–0.3 g of dry fish samples was weighed directly into extraction vessels and 8 mL of extraction solvent was added. Deionized water and water-methanol solution (1:1; 1:2, and 1:3) were used as extraction solvents. Three replicates were carried out for each sample. For ultrasonic extraction using a titanium probe, samples were sonicated from 3 min to 10 min using 60% of the device power. In the case of UB, samples were placed in the ultrasonic bath and sonicated from 15 min to 60 min at 50 °C. In both cases, samples were centrifuged at 6000 rpm for 15 min. However, with use of ME, samples were extracted according to the following program: (1) ramp time 10 min, power 800 W, temperature 80 °C; (2) hold time 15 min, power 800 W, temperature 80 °C; (3) cooling 10 min, power 0 W. Samples containing methanol were evaporated at 60 °C or dried in the air until dryness. The supernatants were then diluted with deionized water. Samples extracted with 100% deionized water were decanted; the residue was washed with deionized water and combined with decanted supernatant. All supernatants were filtered through 0.45 µm membrane syringe filters (VWR International, Radnor, Pensylwania, USA). Samples were frozen before speciation analysis. The extraction procedure was optimized using CRMs: Tuna Fish Tissue BCR-627; Fish Muscle ERM-BB422; Herring Tissue MODAS-3; and Cod Tissue MODAS-5.

## 4. Conclusions

Methods for total arsenic determination and arsenic species determination in freshwater fish samples using ICP-DRC-MS and HPLC/ICP-DRC-MS techniques were developed. Due to the fact that arsenic species in freshwater fish are not extensively studied compared to marine fish, this paper demonstrates results concerning TAs and arsenic species determination in freshwater fish collected from concrete areas located in Poland.

During development of analytical procedures, special emphasis was put on sample preparation steps, especially on extraction. Ultrasonic bath and probe, as well as microwave assisted extraction, were investigated for extraction purposes, but only by using microwave assisted extraction sufficiently high extraction efficiencies were obtained (from 79%). The applied separation conditions achieved full separation of five arsenic species in 8 min. Special attention was paid to the resolution of AsB and As(III) and overlapping of these signals in the presence of high concentration of AsB and trace amounts of As(III). It is known that freshwater fish samples may contain toxic arsenic species, like As(III) and As(V) (sometimes occurring only at trace levels), combined with the presence of AsB (which may be a predominant species), which is problematic to separate and determine. Our experiment revealed that in the presence of about 35 µg L^−1^ (4.5 µg g^−1^) of AsB, it is possible to determine As(III) from the concentration of about 2 µg L^−1^ using the proposed mobile phase. Application of mobile phase containing ammonium carbonate resulted in better separation between AsB and As(III), which enabled the determination of As(III) from the concentration of 1 µg L^−1^. For quantification of the As(III) at a concentration below 1 µg L^−1^, the standard addition method is essential.

In order to assess the applicability of the method, TAs and arsenic species in freshwater fish samples of different species collected from Wielkopolska and Lower Silesia provinces were determined. The predominant species in all freshwater fish samples was AsB. A trace amount of As(V) was stated; this form comprised from 0.72% to 3.34% of TAs but only in samples collected from Lower Silesia province. Results indicate that occurrence of concrete arsenic species do not depend on fish species, but rather depend on area they live in. Investigated fish do not pose a threat to human health. Proposed procedures were validated and provide the reliable data on TAs and arsenic species. We believe these results may be useful for other authors in order to compare their results and for references purposes. Also, in the context of relatively few works existing and conflicting conclusions being presented in literature on arsenic speciation in freshwater fish samples, this work is valuable as it expands knowledge about this kind of fish.

## Figures and Tables

**Table 1:** Total arsenic concentration in certified reference materials of Tuna Fish Tissue BCR-627 and Fish Muscle ERM-BB422 in digested samples (*n* = 3).

	Tuna Fish Tissue BCR-627			Fish Muscle ERM-BB422		
	Mass Fraction [µg g^−1^]	Standard Deviation [µg g^−1^]	*R* [%]	Mass Fraction [µg g^−1^]	Standard Deviation [µg g^−1^]	*R* [%]
^91^AsO^+^/DRC/^74^Ge^+^	5.27	0.23	110	13.59	0.61	107
^91^AsO^+^/DRC/^103^Rh^+^	4.92	0.47	103	13.21	0.17	104
^91^AsO^+^/DRC/-	**4.81**	**0.18**	**100**	**12.99**	**0.56**	**102**
^75^As^+^/-/^74^Ge^+^	4.762	0.084	99	13.79	0.50	109
^75^As^+^/-/^103^Rh^+^	4.611	0.041	96	13.68	0.74	108
^75^As^+^/-/-	4.353	0.040	91	13.67	0.51	108

Certified values of arsenic in: Tuna Fish Tissue BCR-627—Mass fraction ± Uncertainty (4.8 ± 0.3) [µg g^−1^]; Fish Muscle ERM-BB422—Mass fraction ± Uncertainty (12.7 ± 0.7) [µg g^−1^]; *R*—recovery.

**Table:** 

Tuna Fish Tissue BCR-627	H_2_O			H_2_O:CH_3_OH 1:1		
	Mass Fraction [µg g^−1^]	Standard Deviation [µg g^−1^]	*R* [%]	Mass Fraction [µg g^−1^]	Standard Deviation [µg g^−1^]	*R* [%]
^91^AsO^+^/DRC/^74^Ge^+^	4.94	0.67	103	3.23	0.27	67
^91^AsO^+^/DRC/^103^Rh^+^	5.00	0.7	104	3.91	0.32	81
^91^AsO^+^/DRC/-	**4.71**	**0.72**	**98**	3.57	0.21	82
^75^As^+^/-/^74^Ge^+^	4.92	0.75	102	3.87	0.33	81
^75^As^+^/-/^103^Rh^+^	4.72	0.73	103	4.32	0.32	90
^75^As^+^/-/-	5.09	0.58	106	4.677	0.055	97

Certified values of arsenic in: Tuna Fish Tissue BCR 627—Mass fraction ± Uncertainty (4.8 ± 0.3) [µg g^−1^]; *R*—recovery.

**Table:** 

Herring Tissue MODAS-3	H_2_O			H_2_O:CH_3_OH 1:1		
	Mass Fraction [µg g^−1^]	Standard Deviation [µg g^−1^]	*R* [%]	Mass Fraction [µg g^−1^]	Standard Deviation [µg g^−1^]	*R* [%]
^91^AsO^+^/DRC/^74^Ge^+^	7.73	0.66	84	5.16	0.32	56
^91^AsO^+^/DRC/^103^Rh^+^	7.83	0.59	85	6.00	0.21	65
^91^AsO^+^/DRC/-	**8.52**	**0.32**	**92**	6.57	0.36	71
^75^As^+^/-/^74^Ge^+^	7.23	0.12	78	6.14	0.16	66
^75^As^+^/-/^103^Rh^+^	6.96	0.13	75	6.65	0.22	72
^75^As^+^/-/-	8.6	1.3	93	7.82	0.16	85

Certified values of arsenic in: Herring Tissue MODAS-3—Mass fraction ± Uncertainty (9.26 ± 0.81) [µg g^−1^].

**Table 3:** Characteristics of developed analytical procedures for total arsenic and arsenobetaine, arsenite(III), dimethylarsenic acid, monomethylarsenic acid and arsenate(V) determination in freshwater fish samples by inductively coupled plasma mass spectrometry with dynamic reaction cell and high performance liquid chromatography inductively coupled plasma mass spectrometry with dynamic reaction cell, respectively.

Analytical Procedure Parameters	Measurement Result					
	TAs	AsB	As(III)	DMA	MMA	As(V)
Retention time [min]	-	1.8	2.2	2.5	4.6	6.9
Linear range [µg L^−1^]	0.2–50	0.5–10.0	0.5–10.0	0.5–10.0	0.5–10.0	0.5–10.0
Correlation coefficient	0.9999	0.9995	0.9992	0.9994	0.9991	0.9990
L_D_ [µg L^−1^]	0.056	0.13	0.11	0.11	0.14	0.15
L_Q_ [µg L^−1^]	0.17	0.38	0.33	0.34	0.41	0.45
Short-term repeatability [%]	2.2	4.2	4.1	3.3	3.4	3.7
Long-term repeatability [%]	3.6	7.0	5.5	7.2	5.8	4.2
Recovery [%]:						
- for CRM	99	98	-	103	-	-
- samples spiked with 1 µg L^−1^	-	110	95	97	85	116
- samples spiked with 5 µg L^−1^	-	103	97	98	99	105

**Table 4:** Results for As species in certified reference material presented as the average and standard deviations of three replicates of each sample.

**CRM**	**Mass Fraction ± Standard Deviation [µg g^−1^]**				**CR [%]**	
	**AsB**	**DMA**	**∑ As**	**∑ As**		
Fish Muscle ERM-BB422	12.47 ± 0.53	-	12.47 ± 0.53	12.7 ± 0.7	98	
Herring Tissue MODAS-3	8.45 ± 0.40	0.241 ± 0.020	8.69 ± 0.42	9.26 ± 0.81	94	
Cod Tissue MODAS-5	1.102 ± 0.091	0.391 ± 0.038	1.45 ± 0.13	1.64 ± 0.27	89	
	**Mass Fraction ± Uncertainty [µmol kg^−1^]**				**CR [%] AsB**	**CR [%] DMA**
	AsB *	AsB **	DMA *	DMA **		
Tuna Fish Tissue BCR-627	50.77 ± 1.36	52 ± 3	2.07 ± 0.37	2.0 ± 0.3	98	103

CR—column recovery; *—uncertainty expressed as standard deviation, **—certified values for AsB and DMA in CRM.

**Table 5:** Concentration of total arsenic and arsenic species in selected freshwater fish samples from Wielkopolska and Lower Silesia provinces (*n* = 3).

Sample	Province	Mass Fraction ± Standard Deviation [µg g^−1^]					EE [%]	CR [%]
		D TAs	E TAs	E AsB	E As(V)	∑ As Species		
S2 (silver bream)	W	0.0900 ± 0.0061	0.0801 ± 0.0067	0.0752 ± 0.0041	<*L*_D_	0.0752 ± 0.0041	89	94
S3 (silver bream)	W	0.116 ± 0.010	0.1030 ± 0.0070	0.0880 ± 0.0063	<*L*_D_	0.0880 ± 0.0063	89	85
S10 (bream)	W	0.518 ± 0.018	0.509 ± 0.023	0.447 ± 0.013	<*L*_D_	0.447 ± 0.013	98	88
S11 (carp)	W	0.0660 ± 0.0030	0.0622 ± 0.0080	0.0604 ± 0.0041	<*L*_D_	0.0604 ± 0.0041	94	97
S16 (bream)	LS	0.379 ± 0.029	0.299 ± 0.021	0.3008 ± 0.0087	0.0101 ± 0.0010	0.3109 ± 0.0097	79	104
S18 (trout)	LS	4.52 ± 0.12	4.211 ± 0.097	3.87 ± 0.16	0.1337 ± 0.0033	4.00 ± 0.16	93	95
S21 (sturgeon)	LS	5.932 ± 0.057	5.508 ± 0.023	5.23 ± 0.10	0.0379 ± 0.0021	5.27 ± 0.10	93	96
S22 (trout)	LS	4.822 ± 0.067	4.14 ± 0.14	4.16 ± 0.11	0.0570 ± 0.0023	4.22 ± 0.11	86	102

W—Wielkopolska; LS—Lower Silesia; D—digestion; E—extraction; EE—extraction efficiency; CR—column recovery.

**Table 6:** Optimization parameters for separation and determination of TAs and AsB, As(III), DMA, MMA, As(V) using ICP-DRC-MS and HPLC/ICP-DRC-MS techniques.

	Parameter	Setting
HPLC	Instrument	PE series 200 HPLC pump,
		PE series 225 HPLC autosampler
		PE series 200 column oven
	Column	Hamilton PRP-X100
	Elution	isocratic
	Mobile phase	ammonium phosphate, ammonium nitrate
	Concentration of mobile phase	10 mmol L^−1^ (NH_4_)_2_HPO_4_, 10 mmol L^−1^ NH_4_NO_3_
		9.1
	pH of mobile phase	1.0 mL min^−1^
	Mobile phase flow rate	100 µL
	Injection volume	20 °C–25 °C
	Column temperature	
ICP-MS	Instrument	PE Sciex ELAN 6100 DRC II
	RF power	1100 W–1250 W
	Nebulizer gas (Ar) flow rate	0.88 L min^−1^–0.94 L min^−1^
	Auxiliary gas (Ar) flow rate	1.2 L min^−1^
	Plasma gas (Ar) flow rate	15 L min^−1^
	Sampler and skimmer cones	Pt
	Lens voltage	7.0 V–10 V
	Detector mode	Dual
	Data collection mode	^91^AsO, ^75^As
	Scan mode	Peak hopping
	Dwell time	250 ms
	DRC gas (O_2_) flow rate	0.55 L min^−1^
	Rpq	0.45
	Rpa	0
